# Synthetic ALSPAC longitudinal datasets for the Big Data VR project

**DOI:** 10.12688/wellcomeopenres.12441.1

**Published:** 2017-08-30

**Authors:** Demetris Avraam, Rebecca C. Wilson, Paul Burton

**Affiliations:** 1 Data 2 Knowledge Research Group, Institute of Health and Society, Newcastle Biomedical Research Building, Newcastle University, Newcastle upon Tyne, NE4 5PL, UK

**Keywords:** Simulated data, synthetic data, virtual reality, visual analytics, data visualisation, ALSPAC

## Abstract

Three synthetic datasets - of observation size 15,000, 155,000 and 1,555,000 participants, respectively - were created by simulating eleven cardiac and anthropometric variables from nine collection ages of the ALSAPC birth cohort study. The synthetic datasets retain similar data properties to the ALSPAC study data they are simulated from (co-variance matrices, as well as the mean and variance values of the variables) without including the original data itself or disclosing participant information.  In this instance, the three synthetic datasets have been utilised in an academia-industry collaboration to build a prototype virtual reality data analysis software, but they could have a broader use in method and software development projects where sensitive data cannot be freely shared.

## Introduction

In 2015 Wellcome Trust and Epic Games ran a challenge - pairing computer games developers with researchers - to develop visualisation methods in virtual reality (VR) for big biomedical datasets from the following Wellcome Trust funded research projects:

1. A collection of historical medical records from the Casebooks Project
2. Genomics data from the Sanger Institute
3. Cohort data from the ALSPAC study (also known as Children of the Nineties)

University of Bristol researchers were paired with team Luma Pie (comprising Masters of Pie http://www.mastersofpie.com and Lumacode http://www.lumacode.com), who won the challenge with the vARC concept designed to visualise complex cohort data from the ALSPAC study. The Masters of Pie blog records vARC development (part 1, http://www.mastersofpie.com/big-data-vr-challenge/ and part 2 http://www.mastersofpie.com/big-data-vr-challenge-phase-2-update/), and a description of the challenge winning vARC prototype ( http://www.mastersofpie.com/big-data-vr-challenge-winners/).

The value of this emerging technology and its potential applications to e-health and wider use in medicine was recognised by the winning collaboration who continue the development of a VR proof-of-concept biomedical data exploration and visualisation tool under the Big Data VR project using the ALSPAC cohort study as a use case. This project has additionally explored a variety of VR visual analytic methodologies, investigated VR analytics applied to different scales of data and scoped the integration of privacy protecting analytical methods via DataSHIELD ^1^. Findings will be reported in a forthcoming paper.

Due to the nature of the Big Data VR project, it was necessary to use a dataset that could be freely shared across the project team of researchers and games developers, as well as be deployed as an open test dataset for a demo release of the Big Data VR tool. There exist, however, ethical-legal constraints on the open sharing of, or access to, biomedical study data due to concerns around participant privacy and disclosure risk. ALSPAC deploys a rigorous data governance and access policy to protect participant data confidentiality and disclosure. This meant that we could not simply share real ALSPAC data with the developers without going through a potentially lengthy process of formally assessing the bona-fides of every single person in the development team who may need to work with or see the data. Given the very short time scale of the project this was not feasible. However, in order to properly challenge the developers and their evolving tools, and to ensure that the tools would ultimately be useful in a meaningful scientific context, it was nevertheless important that the test datasets closely mirrored real ALSPAC data. To ensure privacy protection, it was therefore necessary to generate synthetic datasets to be used in the project, an approach commonly utilised within the research health data domain ^2^. This paper outlines three synthetic datasets simulated from ALSPAC study data for the purposes of the Big Data VR project.

## Methods

Based at the University of Bristol, ALSPAC (also known as Children of the 90s) studies the health and well-being of pregnancies from the Avon region - with children born between 1991–1992. The whole cohort includes children from original enrolment (phase I recruitment), as well as children invited to join from the age of 7 onwards (from phase II and III recruitment), n = 15445 participants (excluding triplets and quadruplets) at the time of this work. Cohort profiles are described in Boyd et al. ^3^, Fraser et al. ^4^ and the study website contains details of all the data that is available through a fully searchable data dictionary ( http://www.bris.ac.uk/alspac/researchers/data-access/data-dictionary/). The variables from 15445 ALSPAC child participants used for the simulated data generation are outlined in Table 1. They include cardiac measures (i.e. blood pressure and pulse rate) and anthropometric measures (i.e. height, sitting height, weight, bmi, hip and waist circumference) of children visiting different ALSPAC clinics. The age indicated at each clinic is the age of the child at attendance, which is calculated from the date of the visit and the child’s date of birth. All variables used were continuous, except gender which is a binary variable (with 1 indicating male and 2 indicating female). The coverage of these variables at different clinic ages is shown in Table 2, highlighting any variables missing from collection.

**Table 1.** A description of the ALSPAC variables used to generate the simulated datasets.

ALSPAC Variable Name	Description	Simulated Variable Name
kz021	Sex	sex
f7ms010	Height (cm): F@7	height.7
f7ms012	Sitting height (cm): F@7	height.sit.7
f7ms018	Waist circumference (cm): F@7	waist.7
f7ms020	Hip circumference (cm): F@7	hip.7
f7ms026	Weight (kg): F@7	weight.7
f7ms026a	BMI: F@7	BMI
f7sa021	Mean BP systolic: samples F@7	sbp.7
f7sa022	Mean BP diastolic: samples F@7	dbp.7
f7sa023	Mean Pulse: samples F@7	pulse.7
f7003c	Age (months) at Focus @ 7 visit	age.7
f8lf020	Child height (cm): LF, F@8	height.8
f8lf021	Child weight (kg): LF, F@8	weight.8
f8003c	Age (months) at Focus @ 8 visit	age.8
f9ms010	Height (cm): F@9	height.9
f9ms012	Sitting height (cm): F@9	height.sit.9
f9ms018	Waist circumference (cm): F@9	waist.9
f9ms020	Hip circumference (cm): F@9	hip.9
f9ms026	Weight (kg): F@9	weight.9
f9ms026a	BMI: F@9	BMI.9
f9sa021	Mean BP systolic: samples F@9	sbp.9
f9sa022	Mean BP diastolic: samples F@9	dbp.9
f9sa023	Mean Pulse: samples F@9	pulse.9
f9003c	Age (months) at Focus @ 9 visit	age.9
fdms010	Height (cm): F10+	height.10
fdms012	Sitting height (cm): F10+	height.sit.10
fdms018	Waist circumference (cm): F10+	waist.10
fdms026	Weight (kg): F10+	weight.10
fdms026a	BMI: F10+	BMI.10
SBP	Systolic blood pressure_AS	sbp.10
DBP	Diastolic blood pressure_AS	dbp.10
fd003c	Age (months) at F10+ visit	age.10
fems010	Height (cm): F11+	height.11
fems012	Sitting height (cm): F11+	height.sit.11
fems018	Waist circumference (cm): F11+	waist.11
fems020	Hip circumference (cm): F11+	hip.11
fems026	Weight (kg): F11+	weight.11
fems026a	BMI: F11+	BMI.11
fesa021	Mean BP systolic: samples F11+	sbp.11
fesa022	Mean BP diastolic: samples F11+	dbp.11
fesa023	Mean Pulse: samples F11+	pulse.11
fe003c	Age (months) at F11+ visit	age.11
ff2000	M5: Height (cms)	height.12
ff2005	M7: Sitting height (cms)	height.sit.12
ff2020	M11: Waist circumference (cms)	waist.12
ff2620	B8: BP result 1 - systolic	sbp.12
ff2621	B9: BP result 1 - diastolic	dbp.12
ff2622	B10: BP result 1 - pulse	pulse.12
ff0011a	DV: Age of study child at attendance (months)	age.12
fg3100	M5: Height (cms) : TF2	height.13
fg3120	M11: Waist circumference (cms) : TF2	waist.13
fg3130	M15: Weight (Kgs) : TF2	weight.13
fg6120	B15: BP result 1 - systolic : TF2	sbp.13
fg6121	B16: BP result 1 - diastolic : TF2	dbp.13
fg6122	B17: BP result 1 - pulse : TF2	pulse.13
fg0011a	DV: Age of study child at attendance (months) TF2	age.13
fh3000	M5: Height (cms) : TF3	height.15
fh3010	M15: Weight (Kgs) : TF3	weight.15
fh4020	M11: Waist circumference (cms) : TF3	waist.15
fh4030	V6: Sitting height (cms) : TF3	height.sit.15
fh2030	AC18: BP result 1 - systolic : TF3	sbp.15
fh2031	AC19: BP result 1 - diastolic : TF3	dbp.15
fh2032	AC20: BP result 1 - pulse : TF3	pulse.15
fh0011a	DV: Age of study child at attendance (months) TF3	age.15
FJMR020	M5: Height (cms) [F17]	height.17
FJMR022	M15: Weight (kgs) [F17]	weight.17
FJAR020a	dv: Right arm BP mean: systolic	sbp.17
FJAR020b	dv: Right arm BP mean: diastolic	dbp.17
FJAR020c	dv: Right arm BP mean: pulse	pulse.17
FJMR022a	dv: BMI [F17]	bmi.17
FJ003a	Age in months at clinic visit [F17]	age.17

**Table 2.** A summary of data capture in clinics for the respective ALSPAC variables.

Variable (units)	F@7	F@8	F@9	F@10	F@11	TF1	TF2	TF3	TF4
Gender (1 male, 2 female)	yes	yes	yes	yes	yes	yes	yes	yes	yes
Exact Age (months)	yes	yes	yes	yes	yes	yes	yes	yes	yes
Height (cm)	yes	yes	yes	yes	yes	yes	yes	yes	yes
Sitting Height (cm)	yes	NA	yes	yes	yes	yes	NA	yes	NA
Waist Circumference (cm)	yes	NA	yes	yes	yes	yes	yes	yes	NA
Hip Circumference (cm)	yes	NA	yes	NA	yes	NA	NA	NA	NA
Weight (kg)	yes	yes	yes	yes	yes	yes	yes	yes	yes
Systolic Blood Pressure (mmHg)	yes	NA	yes	yes	yes	yes	yes	yes	yes
Diastolic Blood Pressure (mmHg)	yes	NA	yes	yes	yes	yes	yes	yes	yes
Pulse (Beats per minute)	yes	NA	yes	NA	yes	yes	yes	yes	yes
BMI (kg/m2)	yes	NA	yes	yes	yes	NA	NA	NA	yes

Synthetic data was simulated using the statistical programming language R ( 5, version 3.2.3) comprising the following steps with the corresponding R functions noted in line:

### Data cleaning

The ALSPAC dataset described in Table 1 and Table 2 was cleaned by removing all rows with missing values, leaving 1593 observations remaining.

### Standardising continuous variables

Each continuous variable, *x*, was standardised using the the z-score transformation:

$$z=\frac{x–\mu }{\sigma }$$

where *z* denotes the standardised version of the variable, with *µ* and *σ* representing the mean and standard deviation of *x* , respectively (using mean() and sd()). This z-score transformation was used to transform normally distributed data *N* ( *µ*, *σ*) to standard normally distributed data *N* (0, 1).

### Data generation: Continuous variables

It was assumed that the continuous variables (excluding BMI) follow an approximate multivariate normal distribution. Using the pseudo-random multivariate normal generator ( mvrnorm()), three synthetic datasets were generated of observation sizes 15500, 155000 and 1550000 participants. Using the assumption of approximate multivariate normality (without transforming any non-normal data to normal), the synthetic data do not have precisely the same joint and marginal distributions as the original ALSPAC data, but they have very accurate approximations with most variables passing formal tests of normality. The simulated continuous variables were then rescaled back to their original mean and standard deviation by the inverse z-score transformation:

X=Zσ+μ

where *X* and *Z* denote the simulated data for *x* and *z* respectively, with *µ* and *σ* representing (as above) the mean and standard deviation of the real x data.

### Data generation: Binary variables

The simulated gender variable retains the same proportions of males and females as that in the original ALSPAC data set. This was achieved by converting the levels 1-2 (indicating males and females respectively) to 0-1 data and then applying a logistic model for gender regressed ( glm()) on all continuous variables using the original dataset. The estimated coefficients were then used to calculate the linear predictors of the simulated datasets. Then, using the log odds, *y*, from the linear predictors, we have calculated the odds, *p*, that indicate the probability ratio between males and females, using the inverse logit (also known as expit) transformation:

$$p=\frac{\exp(y)}{1+\exp(y)}$$

The simulated binary variable denoting gender in each subject was then generated using the value of *p* in that individual (derived from the expit transformation) as the probability argument in R’s rbinom() function.

### Data generation: BMI variable

The simulated BMI variable was calculated from the simulated values of weight and height for the clinics F@7, F@9, F@10, F@11 and TF4 using the relationship

$$BMI=\frac{weight}{{\left(height/100\right)}^{2}}$$

### Data generation: Age variable

The age at each clinic, initially reported in months, was divided by 12 to represent its values in years. The simulated age variable at each clinic was generated assuming normality and using the rnorm() R function with mean and variance set equal to the actual mean and variance of age at each clinic.

### Dataset validation

Three synthetic datasets were simulated using the methodology described above - with observation size 15,500 participants (simulated.data.1.csv), 155,500 participants (simulated.data.2.csv) and 1,555,000 participants (simulated.data.3.csv). Table 1 shows the data dictionary for these.

The three synthetic datasets have similar properties to the ALSPAC data they are simulated from. This is demonstrated by the close similarity of the estimated means, variances and covariance matrices for the relevant variables in the original ALSPAC dataset and the three synthetic datasets (see Supplementary material). The synthetic datasets contain none of the original data itself.

## Data and software availability

1. The ALSPAC dataset (project number B2506) these synthetic data are simulated from can be obtained from ALSPAC through the standard ALSPAC research proposal and data access policy http://www.bristol.ac.uk/alspac/researchers/access/.
2. The script to generate the three synthetic datasets. https://doi.org/10.5281/zenodo.817502 ^6^
3. The synthetic data described in this paper are available at the University of Bristol data repository, data.bris, at https://doi.org/10.5523/bris.3116aupg8mfgi23pnslu8tulev ^7^

## Ethical statement

Ethical approval for the study was obtained from the ALSPAC Ethics and Law Committee and the Local Research Ethics Committees. A comprehensive list of research ethics committee approval references is available to download at: http://www.bristol.ac.uk/ alspac/researchers/research-ethics/.

## Supplementary material

**Supplementary File 1**: The variable mean for the ALSPAC dataset and the three synthetic datasets.

**Supplementary File 2**: The variable variance for the ALSPAC dataset and the three synthetic datasets.

**Supplementary File 3**: The covariance matrix of the cleaned ALSPAC data (1593 participants) used for simulating the synthetic datasets.

**Supplementary File 4**: The covariance matrix of the synthetic dataset simulated.data.1.csv (15,500 participants).

**Supplementary File 5**: The covariance matrix of the synthetic dataset simulated.data.2.csv (155,500 participants).

**Supplementary File 6**: The covariance matrix of the synthetic dataset simulated.data.3.csv (1,555,500 participants).
