# Supplementary material for: Synthetic ALSPAC longitudinal datasets for the Big Data VR project
Source: Wellcome Open Res. 2017 Aug 30;2:74. [Version 1] doi: 10.12688/wellcomeopenres.12441.1 (PMC5605951; doi:10.12688/wellcomeopenres.12441.1)
Supplement: Peer review history

# Peer review: Referee response for version 1

**DOI:** 10.21956/wellcomeopenres.13472.r25539

**Published:** 2017-11-29

**Authors:** Christopher J. Newby

**Affiliations:** 1 Queen Mary University of London, London, UK

The authors have created a dummy dataset that has similar properties to the real dataset but none of the information governance issues in terms of sharing the data. This will be a great example dataset for teaching purposes as it has no IG restrictions and contains no outliers or missing values. It could also be used for research into data handling, storing or visualisation as suggested in the main body of the article. The data was easily accessible in .CSV files and the files are accompanied by a data dictionary which describes the data well.

I have read this submission. I believe that I have an appropriate level of expertise to confirm that it is of an acceptable scientific standard.

# Peer review: Referee response for version 1

**DOI:** 10.21956/wellcomeopenres.13472.r25537

**Published:** 2017-09-18

**Authors:** Paula J. Robson

**Affiliations:** 1 CancerControl Alberta, Alberta Health Services, Calgary, AB, Canada

This report describes the creation of three simulated datasets using the ALSPAC (Children of the Nineties) cohort. The data are easily accessible, and the provision of the links to the 'Masters of Pie' blog helps provide some interesting context for the work.

 Some very minor points for the authors' consideration:

 It is mentioned that the VR project had a very short time scale - it might be useful to provide some information about the time scale, and how this relates to a typical turnaround time for a formal data request using the usual ALSPAC process. 

 Could the authors provide a little more information to explain why they created three synthetic datasets of different sizes? Why 15500, 155000 and 1550000?

 Table 2 suggests that weight and height were available at all timepoints. However, Table 2 lists 'NA' for BMI at various timepoints - if weight and height were available, why was BMI not derived? 

 Finally, in Supplementary File 2 (variable variance), the variances for the real ALSPAC data are really similar to the variances of the simulated data for most of the variables. The variances for BMI seem to be a little different, with the variances in the simulated datasets being a little higher at all ages across the simulated datasets. Perhaps the authors could comment on this observation?

I have read this submission. I believe that I have an appropriate level of expertise to confirm that it is of an acceptable scientific standard.

# Peer review: Referee response for version 1

**DOI:** 10.21956/wellcomeopenres.13472.r25538

**Published:** 2017-09-11

**Authors:** Alison Park

**Affiliations:** 1 Cohort and Longitudinal Studies Enhancement Resources (CLOSER), Institute of Education, University College London, London, UK

This article describes the process of creating a number of synthetic datasets to underpin a virtual reality visual analysis project. The process is described fully, with working links to appropriate information about the synthetic datasets (and the script used to generate these) and to information about the original ALSPAC data. 

 The importance of the article reflects the fact that it documents an approach to generating synthetic data for use in cases where: a) it is not possible, for ethical and legal reasons, to share the survey or administrative data on which the synthetic data is based; but b) where the synthetic data created needs to mimic the data from which they were derived.

 A few minor editorial comments for the authors to consider:

- The article describes the creation of three synthetic datasets of different sizes, but does not explain the rationale behind the need for these three datasets. More information on this would be useful.
- It would be helpful to introduce the 'Big Data VR' project in the first para of the introduction - it is currently not immediately clear that this is the same thing as the Wellcome & Epic Games 'challenge' (which is the terminology initially used in these introductory sentences).
- The Methods/data cleaning description states that the original ALSPAC dataset was 'cleaned by removing all rows with missing values'. I presume this should read 'by removing all rows with ANY missing values'? 

I have read this submission. I believe that I have an appropriate level of expertise to confirm that it is of an acceptable scientific standard.
